# Synthesis and Application of Novel Magnetic Ion-Imprinted Polymers for Selective Solid Phase Extraction of Cadmium (II)

**DOI:** 10.3390/polym9080360

**Published:** 2017-08-14

**Authors:** Xiaoyan Xu, Mei Wang, Qing Wu, Zhenlin Xu, Xingguo Tian

**Affiliations:** 1 Guangdong Provincial Key Laboratory of Food Quality and Safety, South China Agricultural University, Guangzhou 510640, Guangdong, China; yanzixu_200l@scau.edu.com (X.X.); wm303035@163.com (M.W.); wuqing@scau.edu.cn (Q.W.); jallent@163.com (Z.X.); 2 College of Food Science, South China Agricultural University, Guangzhou 510640, Guangdong, China

**Keywords:** magnetic ion-imprinted polymers, cadmium ion, dual functional monomers, solid-phase extraction

## Abstract

Ion-imprinted polymers (IIPs) have received much attention in the fields of separation and purification. Nevertheless, selectivity of IIPs for trace target ions in complicated matrix remains a challenge. In this work, a cadmium magnetic ion-imprinted polymer (MIIP) was synthesized via surface imprinting, using methacrylic acid and acrylamide as dual functional monomers, vinyltrimethoxysilane as ligand, Fe_3_O_4_@SiO_2_ as support, azodiisobutyronitrile as initiator, and ethylene glycol dimethacrylate as crosslinker. The MIIP was characterized by transmission electron microscopy, infrared spectroscopy, thermal gravimetric analysis, and a vibrating sample magnetometer. The maximum adsorption capacities of the MIIP and magnetic non-imprinted polymer for Cd(II) were 46.8 and 14.7 mg·g^−1^, respectively. The selectivity factors of Pb(II), Cu(II), and Ni(II) were 3.17, 2.97, and 2.57, respectively, which were greater than 1. The adsorption behavior of Cd(II) followed the Freundlich isotherm and a pseudo second order model. The MIIP was successfully used for the selective extraction and determination of trace Cd(II) in representative rice samples. The limit of detection and recovery of the method was 0.05 µg·L^−1^ and 80–103%, respectively, with a relative standard deviation less than 4.8%. This study shows that MIIP provides an attractive strategy for heavy metal detection.

## 1. Introduction

Cadmium is one of the most toxic heavy metals and causes serious harm to human health via the food chain [1,2]. The International Agency for Research on Cancer classified cadmium as a human carcinogen [3]. Therefore, developing reliable methods for the removal and determination of cadmium in food is important. A wide range of analytical techniques to evaluate cadmium(II) in the spiked or real samples have been reported in recent years, including atomic absorption spectrometry (AAS) [4], inductively coupled plasma mass spectrometry (ICP-MS) [5], electrochemical sensor [6]. Among them, atomic absorption spectrometry has been popular because of its simplicity, easy operation, and low cost [7]. However, its low sensitivity in trace analysis of complex systems largely limits its applications because of the matrix interferences [8]. Therefore, a suitable separation and preconcentration step such as solid-phase extraction is necessary for concentrating the target analytes from real samples [9].

Ion imprinting is a process in which co-polymerization has been performed between functional and cross-linking monomers in the presence of a target ion (the imprint ion) [10]. Extraction of the template molecules leaves a predetermined arrangement of ligands and a tailored binding pocket [11]. Now, ion-imprinted polymers (IIPs) are becoming promising solid-phase extraction (SPE) sorbents and have gained much attention because of their outstanding advantages such as high selectivity, large adsorption capacity, and good reusability [12,13]. Ghorbani et al. [14] successfully synthesized Pd(II) ion-imprinted polymers for selective extraction and determination in food and environmental samples. A Ni(II) ion-imprinted polymer prepared by Saraji et al. [15] was employed as a sorbent in a solid-phase extraction column to preconcentrate trace nickel with satisfactory results. However, complicated post-processing, such as filtration and centrifugation, are required when applying these ion-imprinted polymers [16]. If ion-imprinted polymer materials are chemically grafted onto Fe_3_O_4_ nanoparticles, which have unique magnetic properties and high surface area, easy separation and selective extraction can be achieved with the application of an external magnetic field. Previously published papers have shown a successful combination of ion-imprinted polymers and Fe_3_O_4_, and superior performance from magnetic imprinted polymers was reported compared with that of imprinted polymers without magnetic nanoparticles attached [17,18,19].

Despite the emerged interests in magnetic ion imprinting techniques, single functional monomers are widely and generally used for preparing imprinted polymers and thereby result in low selectivity. Thus, it has become quite imperative to develop new functional monomers and/or utilize dual/multiple functional monomers for improving the selective recognition capabilities and adsorption capacities of IIPs [20]. In the present work, a novel magnetic ion-imprinted polymer (MIIP) for Cd(II) was synthesized using dual functional monomers of MAA and acrylamide (AM) by surface imprinting. In addition, the characterization, kinetics, isotherm model of Cd(II) adsorption process, selectivity, and application of this new magnetic sorbent are discussed in this study.

## 2. Materials and Methods

### 2.1. Reagents and Chemicals

Cd, Pb, Cu, and Ni reference solutions were supplied by Shanghai Macklin Biochemical Co., Ltd. (Shanghai, China), with a minimum 99% purity. All other reagents and chemicals including FeCl_3_·6H_2_O, FeCl_2_·4H_2_O, PEG200, CdCl_2_·5/2H_2_O, tetraethyl orthosilicate, vinyltrimethoxysilane, hexadecyl trimethyl ammonium bromide, methacrylic acid, acrylamide, ethylene glycol, dimethacrylate, and azodiisobutyronitrile were in analytical grade and purchased from Aladdin Industrial Corporation (Shanghai, China). Cd standard solution (100 mg·L^−1^) was prepared by diluting a reference solution in 2% nitric acid solution. All of the solutions were stored in the fridge (4 °C) and allowed to reach ambient temperature prior to use. The water used was ultrapure (18.3 MΩ·cm resistivity).

### 2.2. Characterization

An AA800 graphite furnace atomic absorption spectrometer (Perkin Elmer, Shelton, CT, USA) was used to determine the concentration of metal ions (Cd, Ni, Cu, and Pb) in aqueous solution. The instrumental parameters were those recommended by the manufacturer. Hollow cathode lamps were used as the radiation sources. The wavelengths selected were 228.8, 232.0, 324.8, and 283.3 nm for Cd, Ni, Cu, and Pb, respectively. A 780 digital pH Meter (Metrohm, Herisau, Switzerland), equipped with a combined Ag/AgCl glass electrode was used for the pH adjustments at room temperature. Fourier transform infrared (FT-IR) spectra (4000–500 cm^−1^) were recorded using a Vertex 70 spectrometer (Bruker, Ettlingen, Germany) with KBr pellets and a resolution of 0.4 cm^−1^. Transmission electron microscope (TEM) images were acquired on a JEM-2100F instrument (JEOL, Peabody, MA, USA). Thermal gravimetric analysis (TGA) was performed on a TG209 F1 Libra instrument (Netzsch, Hanau, Germany) under argon atmosphere at a heating rate of 10 °C/min. Magnetic characteristics of Fe_3_O_4_ and MIIP were measured with an EZ7 vibrating sample magnetometer (MicroSense, Lowell, MA, USA) at 300 K.

### 2.3. Preparation of Vinyl-Functionalized Fe_3_O_4_ Nanoparticles

Fe_3_O_4_ nanoparticles were synthesized by the method of titration hydrolysis with minor modification [21]. Briefly, 0.02 mol FeCl_3_·6H_2_O and 0.012 mol FeCl_2_·4H_2_O were dissolved in 100 mL of deionized water under a nitrogen atmosphere. This solution was subsequently heated to 80 °C by a magnetic stirrer, followed by the addition of 15 mL of NH_3_·H_2_O (32% solution) and 2 mL of PEG200 sequentially. The resultant Fe_3_O_4_ nanoparticles were separated by a magnet, washed three times with ethanol and ultrapure water, and dried in a vacuum oven at 60 °C overnight.

Vinyl modified Fe_3_O_4_ nanoparticles were performed as previously reported [22,23]. Fe_3_O_4_ nanoparticles (0.5 g), 50 mL of anhydrous ethanol and 10 mL of NH_3_·H_2_O were suspended in 100 mL of deionized water, and 2 mL of tetraethyl orthosilicate was added to the mixture dropwise. The Fe_3_O_4_@SiO_2_ was obtained under stirring at room temperature after 6 h. The Fe_3_O_4_@SiO_2_ was dispersed in 100 mL of the mixture of water and ethanol (1:5, *v/v*). Ammonia water (10 mL) containing 2 g of hexadecyl trimethyl ammonium bromide, 4 mL of tetraethyl orthosilicate, and 3.7 mL of vinyltrimethoxysilane were added into the above mixture under vigorous stirring. After reaction at ambient temperature for 6 h, the resultant products were refluxed in acetone solution at 80 °C for 24 h to remove the hexadecyl trimethyl ammonium bromide.

### 2.4. Preparation of Magnetic Cadmium Ion-imprinted Polymer

**Figure 1:**
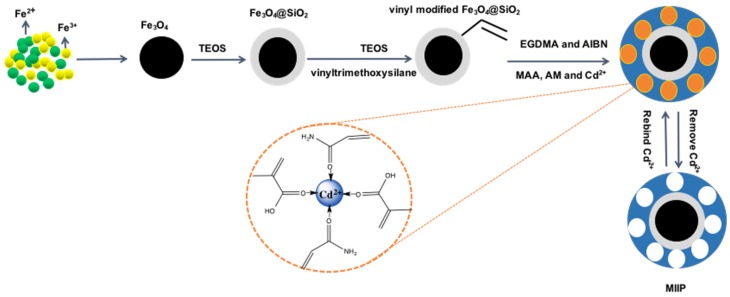
Schematic diagram for the preparation of magnetic ion-imprinted polymer (MIIP).

The preparation of magnetic cadmium ion-imprinted polymer is schematically shown in Figure 1. Briefly, 0.5 mmol of CdCl_2_·5/2H_2_O, 1.5 mmol of methacrylic acid, and 0.5 mmol of acrylamide were dispersed into a mixture solution of acetonitrile (40 mL) and water (10 mL) under stirring at room temperature for 90 min. Then 0.5 g of vinyl-modified Fe_3_O_4_@SiO_2_, 1.98 g of ethylene glycol dimethacrylate and 0.15 g of azodiisobutyronitrile were added consecutively. The polymerization reaction was performed at 85 °C with 10 h of stirring to obtain particles with a highly crosslinked structure. The product was separated with an external magnetic force, washed with ethanol, and oven-dried at 65 °C for 6 h. The template was removed by 2.0 M of HCl. For comparison, magnetic non-imprinted polymer (MNIP) was also prepared using an identical procedure in the absence of the template (cadmium ions).

### 2.5. Adsorption Experiments

The adsorption of Cd(II) from aqueous solutions was investigated in batch experiments. The effect of pH on the adsorption capacity of the MIIP sorbents was studied with 20 mL of 70 µg·mL^−1^ Cd(II) solutions in the pH range 1.0–6.3. The pH of the suspensions was changed using 0.1 M HCl or 0.1 M NH_3_·H_2_O. The static adsorption rates were studied with 20 mL of 70 µg·mL^−1^ Cd (II) solutions at pH 6.0 from 5 to 70 min. To measure the maximum adsorption capacity of MIIP and MNIP, the obtained MIIP (20 mg) and MNIP (20 mg) were added with 20 mL Cd(II) solutions containing different initial concentrations (30–80 µg·mL^−1^) at a constant temperature of 25 °C for 35 min. After complete adsorption, the adsorbent was collected with an external magnetic force, and the supernatant was introduced into the graphite furnace atomic absorption spectrometer for determining Cd(II) concentration. To assess the reusability of MIIP, 20 mg MIIP was added into cadmium standard aqueous solution (70 µg·mL^−1^) for a multiple adsorption–elution–adsorption process under the same conditions. The amount of Cd(II) bound by the polymer was calculated by the following equation:

(1) $$Q=\frac{\left(C_{0}-C_{e}\right)\times V}{W}$$

where *Q* represents the amount of Cd(II) adsorbed (mg·g^−1^), *C*_0_ and *C*_e_ are the initial and equilibrium concentration (µg·mL^−1^) of Cd(II), respectively, *V* is the volume of cadmium standard solution (mL), and *W* is the mass of sorbent (g). All the tests were performed in triplicate.

To investigate the mechanism of the adsorption process, the Freundlich and Langmuir models were applied to fit the adsorption data. The Freundlich and Langmuir adsorption isothermal equations are as follows [24,25]:

(2) $$\mathrm{Freundlich}\;\mathrm{model}\;\ln Q_{e}=\ln K_{f}+\frac{1}{n}\ln C_{e}$$

(3) $$\mathrm{Langmuir}\;\mathrm{model}\;\frac{C_{e}}{Q_{e}}=\frac{C_{e}}{Q_{m}}+\frac{1}{Q_{\max}k_{b}}$$

where *Q*_e_ is the equilibrium adsorption capacity (mg·g^−1^), *C*_e_ is the equilibrium concentration, *K*_f_ and *n* are the Freundlich constants related to the adsorption capacity and intensity, respectively, *Q*_max_ represents the maximum adsorption capacity, and *K*_b_ is the Langmuir constant representing the affinity between the solute and adsorbent. To further understand the mechanism of the adsorption process, two different kinetic models (Laguerre pseudo-first-order and pseudo-second-order kinetic models) were used to fit the kinetic data. The two kinetic models for the adsorption of solid/liquid system are as follows [26,27]:

(4) $$\mathrm{Pseudo}-\mathrm{first}-\mathrm{order}\;\mathrm{model}\;-\ln\left(1-F\right)=k_{a}t$$

(5) $$\mathrm{Pseudo}-\sec\mathrm{ond}-\mathrm{order}\;\mathrm{model}\;\frac{t}{Q_{t}}=\frac{1}{k_{b}Q_{e}^{2}}+\frac{1}{Q_{e}}$$

where *F* equals to *Q*_t_/*Q*_e_, *Q*_t_ and *Q*_e_ are the adsorption capacities of Cd(II) at time *t* and at equilibrium, respectively, and *k*_a_ and *k*_b_ are the first order and second order rate constants, respectively.

The selective adsorption experiments of Pb(II), Cu(II), and Ni(II) ions with respect to Cd(II) ions were conducted using MIIP or MNIP sorbents. Briefly, 20 mg of MIIP and MNIP sorbents were added to 20 mL of a binary mixture containing 20 mg·mL^−1^ Cd(II)/Pb(II), Cd(II)/Cu(II), or Cd(II)/Ni(II) at pH 6.0 for 35 min. The imprinting factor (α) and selectivity factor (β) for Cd(II) ions relative to competing ions were calculated from the following equations:

(6) $$\alpha =\frac{Q_{A}}{Q_{B}}$$

(7) $$\beta =\frac{\alpha_{1}}{\alpha_{2}}$$

where *Q*_A_ and *Q*_B_ represent adsorption capacities of metal ions onto MIIP and MNIP, respectively, and *α*_1_ and *α*_2_ are imprinting factors of Cd(II) and other ions.

### 2.6. Real Sample Pretreatment

To evaluate the practicability of MIIP for Cd(II) extraction from real samples with different matrices, rice 1 (Hunan, China), rice 2 (Heilongjiang, China), and rice 3 (Shandong, China) were selected. Because there were trace Cd(II) in the three rice samples, 0.5, 1, and 2 ng·mL^−1^ Cd(II) standard solutions were also spiked in the above rice samples.

The three spiked rice samples were subjected to acid digestion according to the previously published method [28]. Afterwards, 20 mL of digested solution was transferred into a 50 mL conical flask to form a dark cloudy suspension. Then, MIIP sorbent (30 mg) was added into the sample solution after adjusting the pH to neutral. Extraction was achieved by shaking the sample solution for 35 min. The sorbent was separated by a magnet, and the solution became clear. The sorbent was eluted with 2 M HCl. After 8 h, the supernatant was obtained, and the concentration of Cd(II) ion was determined by graphite furnace atomic absorption spectrometry.

## 3. Results

### 3.1. Characterization

**Figure 2:**
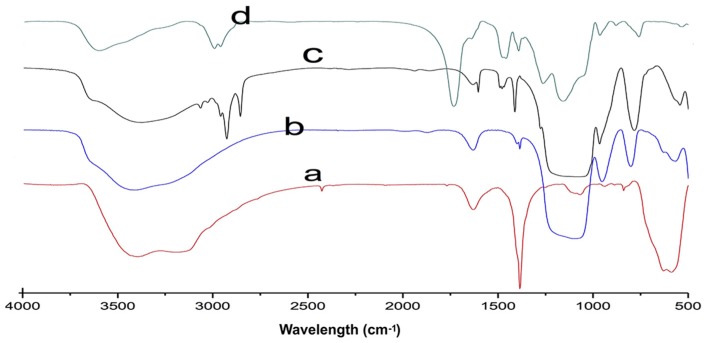
Fourier transform infrared (FT-IR) spectra of Fe_3_O_4_ (**a**), Fe_3_O_4_@SiO_2_ (**b**), vinyl modified Fe_3_O_4_@SiO_2_ (**c**) and MIIP (**d**).

The infrared spectra of Fe_3_O_4_, Fe_3_O_4_@SiO_2_, Fe_3_O_4_@SiO_2_–C=C, and MIIP are displayed in Figure 2. The adsorption peak at 589 cm^−1^ observed in all samples can be assigned to Fe–O stretching vibrations, which indicated the presence of Fe_3_O_4_. These peaks intensity diminished as ion-imprinted polymer was layered onto Fe_3_O_4_ [29]. The peaks of Fe_3_O_4_@SiO_2_ at 1100, 953, and 801 cm^−1^ corresponded to the Si–O–Si, Si–O–H, and Si–O stretching, respectively, and indicated that the silica was modified onto the surface of Fe_3_O_4_ successfully. The peaks at 1603, 3063, and 3026 cm^−1^ coming from C=C stretching suggested that the vinyl groups were grafted onto the surface of Fe_3_O_4_@SiO_2_. The peak of MIIP at 1732 cm^−1^ was the characteristic group frequencies of the C=O stretching vibration, and the appearance of the stretching vibrations at 1263 cm^−1^ belonging to C–N–H indicated that methacrylic acid and acrylamide were both involved in the imprinted polymerization.

**Figure 3:**
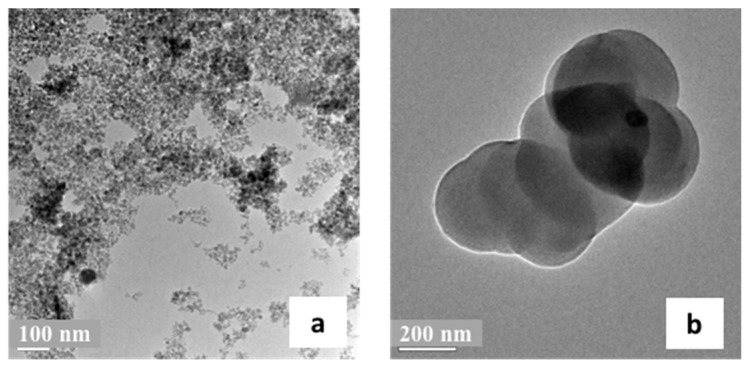
Transmission electron microscopy (TEM) images of Fe_3_O_4_ (**a**) and MIIP (**b**).

Appropriate MIIP shell thickness enables easy access for the target ions and leads to higher adsorption capacity [30]. Figure 3 showed the morphology and microstructure of the Fe_3_O_4_ and MIIP. Magnetic Fe_3_O_4_ particles were spherical and uniform in size, with an average diameter of 20 nm. After polymerization, the Fe_3_O_4_ particles were coated with a thick layer, and the surface was rough and irregular. The diameter of MIIP was larger compared with that of Fe_3_O_4_ and the thickness of the MIIP was about 100 nm. The TEM micrograph observations confirmed that the imprinted layer was successfully immobilized on the surface of Fe_3_O_4_ particles.

**Figure 4:**
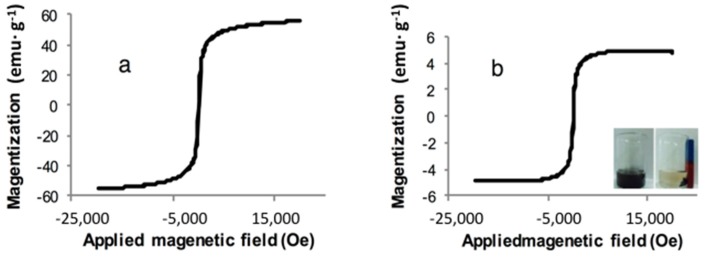
Magnetization hysteresis loops of Fe_3_O_4_ (**a**) and MIIP (**b**).

The magnetic hysteresis loops of Fe_3_O_4_ and MIIP are illustrated in Figure 4. The two loops showed similar shapes and coercive force, which indicated that both of them were superparamagnetic in nature. The saturation magnetization of Fe_3_O_4_ was 55.6 emu·g^−1^. In comparison, the MIIP showed a lower saturation magnetization, which was 4.88 emu·g^−1^, suggesting the formation of the polymer layer on the surface of Fe_3_O_4_@SiO_2_. Therefore, the magnetic separation of MIIP can be achieved with an external magnetic force.

**Figure 5:**
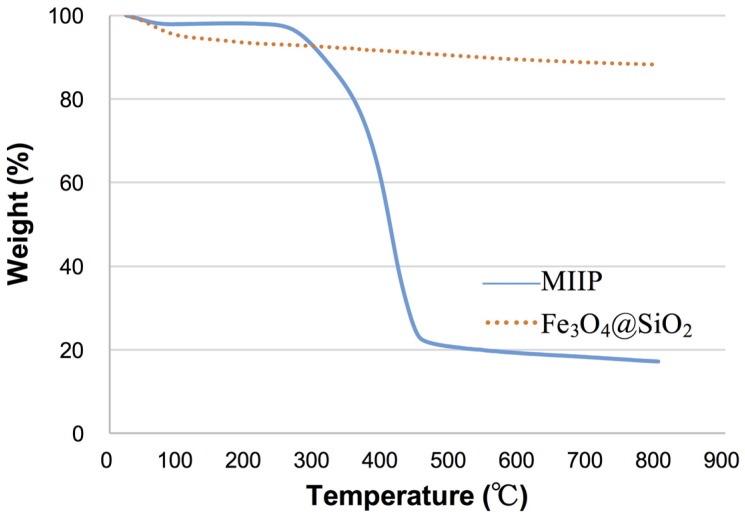
Thermal gravimetric analysis (TGA) curves of Fe_3_O_4_@SiO_2_ and MIIP.

The thermogravimetric curves of MIIP and Fe_3_O_4_@SiO_2_ have been established from room temperature (25 °C) to 800 °C. As shown in Figure 5, Fe_3_O_4_@SiO_2_ exhibited weight loss of less than 16% during the whole process. MIIP had a slight weight loss of about 5% up to 300 °C, which might be assigned to the physical evaporation of H_2_O. The other loss (about 80%) of mass of MIIP occurred between 300 and 600 °C, and was attributed to the thermal decomposition of the organic component in the MIIP. The above results indicate that there is a thin imprinted layer on the surface of Fe_3_O_4_, which is in agreement with the observations from TEM and magnetism analysis. It also demonstrates that the sorbent prepared in this study has a good thermal stability, so it can be used in a wide temperature range.

### 3.2. Adsorption Properties

#### 3.2.1. Adsorption Capacity

**Figure 6:**
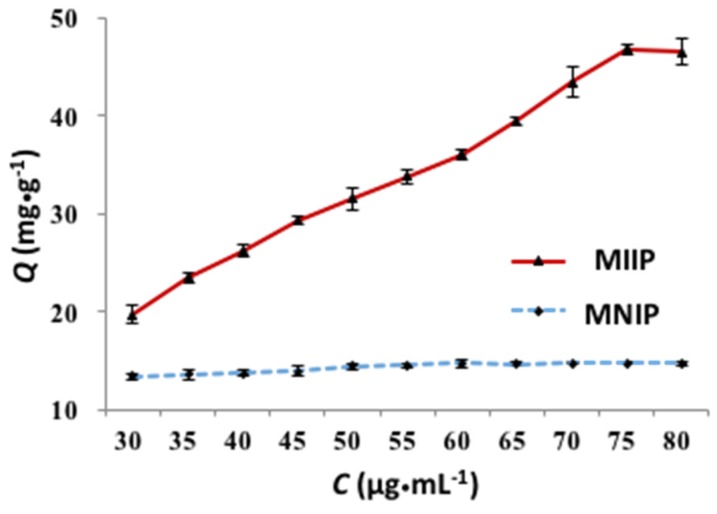
The effect of initial concentration on the adsorption capacity of MIIP and MNIP.

**Figure 7:**
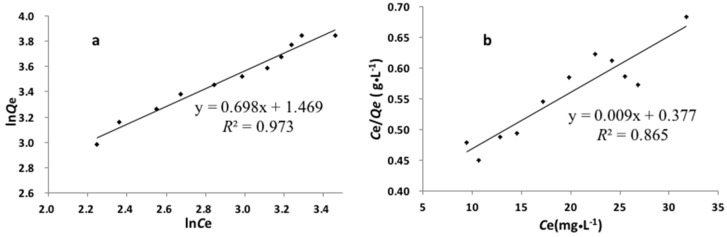
The fitting curves of Freundlich (a) and Langmuir (b) adsorption isotherm of MIIP.

Adsorption capacity is an important indicator for the evaluation of the sorbent. Figure 6 showed the effect of the initial concentration of Cd(II) ion on the adsorption capacity. The adsorption amount of MIIP and MNIP increased with the increase of cadmium ion solution concentration within 30–80 µg·mL^−1.^ The adsorption capacity of MIIP was higher than that of the MNIP under the same experimental concentration, suggesting that Cd(II)-MIIP had imprinted sites because of template ions added during synthesis. The maximum static adsorption capacities of the MIIP and MNIP for Cd(II) ion were 46.80 and 14.70 mg·g^−1^, respectively. The fitting results for the Langmuir and Freundlich isotherms are shown in Figure 7. Compared with the Langmuir model, the Freundlich model was more suitable because of the value of the correlation coefficients (*R*^2^), which suggests that the adsorption of Cd(II) ion onto the MIIP was a multilayer adsorption, and the adsorption occurred on a heterogeneous surface with different active sites. 

#### 3.2.2. Adsorption Kinetics

**Figure 8:**
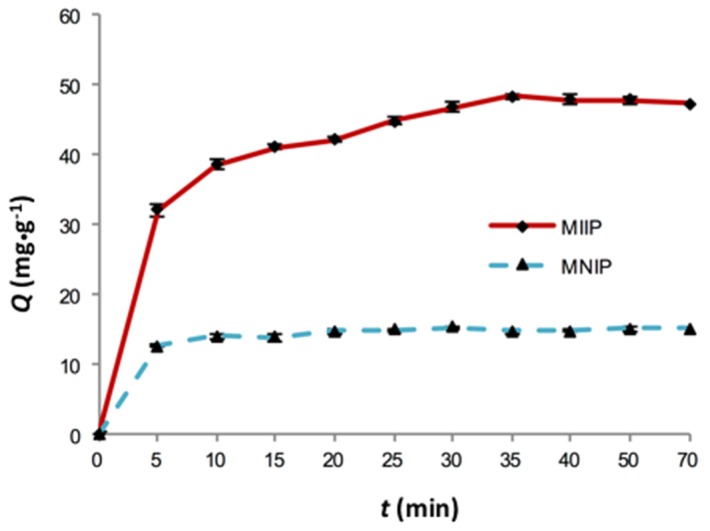
The adsorption dynamics curves of the MIIP and MNIP.

The effect of contact time on the adsorption capacity of Cd(II) ion on the MIIP in aqueous solution at pH 5.0 at room temperature is presented in Figure 8. The amount of Cd ion uptake increased rapidly with the increase of contact time during the first 5 min. From 5 to 35 min, the adsorption amount increased slowly, and a plateau was reached with equilibrium. These results show that there were a large number of specific binding sites on the sorbent surface, which were available for the adsorption of Cd(II) at the early stage. Gradually, the specific binding sites were completely occupied. Furthermore, the adsorption kinetic curve of MNIP was similar to that of MIIP except that the saturated adsorption amount was lower. This difference in capacity was because the cavities left in the MIIP were complementary in size and shape to the template ion, whereas there were no imprinted sites in the MNIP. 

**Figure 9:**
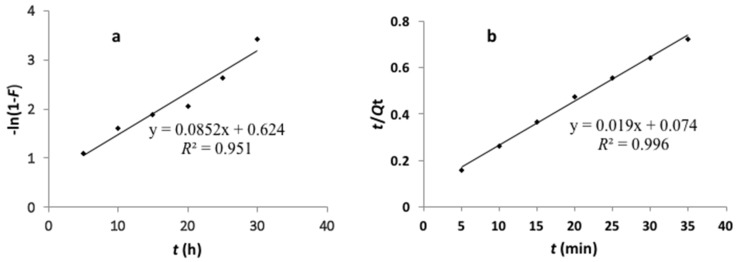
The linear plots of pseudo-first-order (**a**) and pseudo-second-order; (**b**) kinetic model.

The corresponding linear regression correlation coefficients, *R*^2^, were calculated from the linear plots of Equations (4) and (5) and are shown in Figure 9. The Lagergren pseudo-first-order kinetic model showed an *R*^2^ average of 0.951. However, the pseudo-second-order model showed a higher *R*^2^ value (more than 0.99). In addition, the calculated *Q*_e_ values also agreed with the experimental data in the case of pseudo-second-order kinetics. The above results indicated that the pseudo-second-order kinetic model was more suitable to predict the adsorption process of Cd(II) onto MIIP.

#### 3.2.3. Selectivity Adsorption

**Figure 10:**
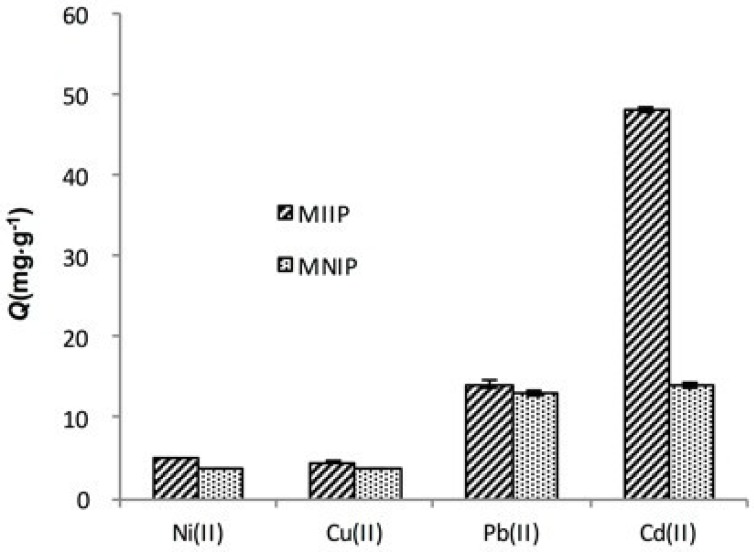
The adsorption capacities of Cd(II), Pb(II), Cu(II), and Ni(II) onto the MIIP and MNIP.

Pb(II), Cu(II), and Ni(II) ions were used as competitive metal ions because of their similar charge and ionic radius with Cd(II). Table 1 showed that the imprinting factors of Cd(II), Pb(II), Cu(II), and Ni(II) were 3.42, 1.08, 1.15, and 1.33, respectively. The selectivity factors of Pb(II), Cu(II), and Ni(II) were 3.17, 2.97, and 2.57, respectively. Figure 10 showed that MIIP exhibited a higher selectivity toward Cd(II) than other ions despite their similar size and charge in solution. Therefore, the high affinity, because of using template ions during imprinting, enabled the MIIP sorbent to extract Cd(II) selectively in the presence of other metal ions.

### 3.3. Effect of Sample pH

**Figure 11:**
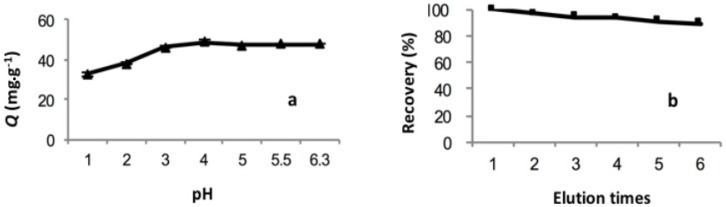
Effect of pH (**a**) and elution times; (**b**) on the adsorption capacity of MIIP.

pH is one of the most important parameters for the adsorption performance of MIIP. The influence of different pH values in the range of 1.0–6.3 on the adsorption capacity of MIIP was studied, and the results are presented in Figure 11a. The results showed that the adsorption capacity increased quickly with the increase of pH value from 1.0 to 4.0, and then remained constant in the pH range of 4.0–6.3. Because the positive charge on the surface of MIIP protonates binding sites, the affinity between MIIP and Cd(II) was weakened at low pH values, and a decrease in adsorption capacity was observed [26]. Thus, the optimum pH was 6.0.

### 3.4. Reusability

The reusability and stability of MIIP were studied by eluting and reusing the same sorbent, and the adsorption results are presented in Figure 11b. As shown in Figure 11b, during the six adsorption–desorption cycles, there was no significant decrease in the adsorption capacity for Cd (II), and the recovery was higher than 90%. The above results indicated that MIIP showed a stable regeneration adsorption efficiency and can be used repeatedly.

### 3.5. Analytical Performance of the Method

As shown in Table 2, the recoveries of Cd(II) ions from the real and spiked samples varied in the range of 80–103%, and the relative standard deviation was less than 4.8%. The limit of detection, defined as *C*_LOD_ = 3 *S*_b_/*m*, where *S*_b_ is the standard deviation of seven replicate blank signals and *m* is the slope of the linear section of calibration curve after preconcentration for a sample volume of 50 mL, was 0.05 µg·L^−1^. In comparison with other reported Cd((II) IIPs for SPE extraction (Table 3), the proposed method shows comparable results in terms of analytical performance. The results support that the synthesized MIIP is a selective and reliable sorbent suitable for preconcentration and determination of Cd(II) ions at trace levels in real samples.

## 4. Conclusions

The novel magnetic ion-imprinted polymers were successfully prepared with the utilization of dual functional monomers and were used as sorbents for the selective separation and preconcentration of trace Cd(II) ions. The MIIPs possessed high adsorption capacity and selectivity toward the template ion. The binding experimental data fit well into the Freundlich isotherm and match well with the pseudo-second-order kinetic model. After the sixth cycle of the adsorption–desorption process, the recovery remained higher than 90% that in the first cycle. Furthermore, the prepared sorbent possessed high adsorption capacity and selectivity toward the template ion. Good recoveries and a low limit of detection were obtained in the analysis of real rice samples. Because of the performance demonstrated in the present study, MIIP is an excellent candidate for the selective determination of Cd(II) in food and environmental samples.

## Figures and Tables

**Table 1:** Selectivity of MIIP towards different metal ions.

Metal ions	*Q*_A_ (mg·g^−1^)	*Q*_B_ (mg·g^−1^)	α	β
Cd(II)	48.13	14.07	3.42	-
Pb(II)	14.13	13.13	1.08	3.17
Cu(II)	4.41	3.83	1.15	2.97
Ni(II)	4.95	3.72	1.33	2.57

**Table 2:** The analysis results of Cd(II) ions in three spiked rice samples.

Sample	Added ng·mL^−1^	Found ng·mL^−1^	Recovery (%)	RSD (%)
Rice 1	0.5	0.41 ± 0.09	82	3.86
	1	0.86 ± 0.11	86	1.60
	2	1.87 ± 0.16	94	2.70
Rice 2	0.5	0.49 ± 0.10	80	4.76
	1	0.94 ± 0.13	93	2.64
	2	2.06 ± 0.15	103	2.00
Rice 3	0.5	0.40 ± 0.06	80	3.76
	1	0.91 ± 0.10	91	1.72
	2	1.80 ± 0.12	90	1.51

**Table 3:** Comparison of analytical performance of different cadmium(II)-imprinted polymers used for SPE extraction.

Monomer or ligand	Detection system	Adsorption capacity (mg·g^−1^)	Selectivity factor			LOD (µg·L^−1^)	RSD (%)	Reference
			Pd	Cu	Ni			
2-VP ^a^	ICP-OES	16.52	N.R.	N.R.	N.R.	0.14	2.6	[31]
4-VP ^b^	FAAS	0.48	N.R.	N.R.	N.R.	0.11	2.9	[7]
ATU ^c^	ICP-AES	38.30	9.46	2.86	6.42	N.R.	N.R.	[32]
TCPTS ^d^	FAAS	44.7	2.44	3.64	N.R.	N.R.	N.R.	[33]
MAH ^e^	ICP-MS	13.8	6.0	38.5	3.5	0.004	3.2	[5]
MAA ^f^,AM ^g^	GFAAS	46.8	3.17	2.97	2.57	0.05	4.8	This work

N.R. represents no reported; ^a^ 2-Vinylpyridine; ^b^ 2-Vinylpyridine; ^c^ Allyl thiourea; ^d^ 3-thiocyanatopropyltriethoxysilane; ^e^ *N*-methacryloly-(*L*)-histidine; ^f^ Methacrylic acid; ^g^ Acrylamide.
